# The roles of RACK1 in the pathogenesis of Alzheimer's disease

**DOI:** 10.7555/JBR.37.20220259

**Published:** 2024-02-27

**Authors:** Wenting He, Xiuyu Shi, Zhifang Dong

**Affiliations:** Pediatric Research Institute, Ministry of Education Key Laboratory of Child Development and Disorders, National Clinical Research Center for Child Health and Disorders, Chongqing Key Laboratory of Child Neurodevelopment and Cognitive Disorders, Children's Hospital of Chongqing Medical University, Chongqing 400014, China

**Keywords:** RACK1, Alzheimer's disease, PKC, amyloid-β, synaptic plasticity, neuroinflammation

## Abstract

The receptor for activated C kinase 1 (RACK1) is a protein that plays a crucial role in various signaling pathways and is involved in the pathogenesis of Alzheimer's disease (AD), a prevalent neurodegenerative disease. RACK1 is highly expressed in neuronal cells of the central nervous system and regulates the pathogenesis of AD. Specifically, RACK1 is involved in regulation of the amyloid-β precursor protein processing through α- or β-secretase by binding to different protein kinase C isoforms. Additionally, RACK1 promotes synaptogenesis and synaptic plasticity by inhibiting N-methyl-D-aspartate receptors and activating gamma-aminobutyric acid A receptors, thereby preventing neuronal excitotoxicity. RACK1 also assembles inflammasomes that are involved in various neuroinflammatory pathways, such as nuclear factor-kappa B, tumor necrosis factor-alpha, and NOD-like receptor family pyrin domain-containing 3 pathways. The potential to design therapeutics that block amyloid-β accumulation and inflammation or precisely regulate synaptic plasticity represents an attractive therapeutic strategy, in which RACK1 is a potential target. In this review, we summarize the contribution of RACK1 to the pathogenesis of AD and its potential as a therapeutic target.

## Introduction

Alzheimer's disease (AD) is a neurodegenerative disease characterized by the buildup of toxic amyloid plaque and intracellular neurofibrillary tangles (NFTs), resulting in a progressive loss of cognitive function and memory. Current leading hypotheses on the pathogenesis of AD include the cholinergic hypothesis, the amyloid cascade hypothesis, and the neuroinflammation hypothesis. The earliest studies of the disease focused on neurochemical analysis and proposed the cholinergic hypothesis. In the brains of Alzheimer's patients, the choline transported to synaptosomes is reduced by 50%–80%. Meanwhile, the loss of cholinergic neurons and synapses may lead to cognitive impairment and neurodegeneration^[1]^. The amyloid cascade hypothesis states that the accumulation of amyloid-beta (Aβ) peptides produced by Aβ precursor protein (AβPP or APP) amyloidogenic process is an important cause of AD. The released Aβ peptides produced by APP with β-secretase and γ-secretase cleavage are toxic to cells, causing cell death and promoting tau hyperphosphorylation, followed by the formation of intracellular NFTs^[2]^. The accumulation of amyloid plaques initially occurs in the frontal cortex and then spreads throughout the cortex with the progression of the disease, and the accumulation of NFTs becomes an important biomarker of AD^[3]^. In addition, neuroinflammation is also a key pathogenic factor in AD, as the activated microglia are a typical pathophysiological feature of AD and other neurodegenerative diseases^[4]^. During AD progression, microglial activation leads to the production and release of abundant pro-inflammatory cytokines, including interleukin (IL)-1β, IL-6, IL-18, and tumor necrosis factor-alpha (TNF-α)^[5]^. The upregulation of these proinflammatory cytokines plays a complex role in both neuroprotection and neurodegeneration. Extracellular Aβ deposition activates innate immunity by binding to pattern recognition receptors, leading to numerous inflammatory mediators (*e.g.*, nitric oxide and reactive oxygen species) and cytokines (*e.g.*, IL-1β, IL-10, IL-33, and TNF-α), and contributing to the development of AD^[4]^. Furthermore, TNF-α stimulates γ-secretase activity, resulting in an increased synthesis of Aβ peptides and a further increase in TNF-α release^[5]^. Together, these hypotheses interpret the symptoms and pathology of AD complementarily.

One member of the tryptophan-aspartate (WD) repeat family is the receptor for the activated C kinase 1 (RACK1) that generates signaling complexes by binding to several group members, because of its seven internal WD40 repeats that form β-propeller blades^[6]^. RACK1 acts as a scaffolding protein for different kinases and membrane receptors, transporting proteins to their sites of action, facilitating crosstalk between different signaling pathways, or recruiting other proteins into complexes. Therefore, RACK1 is a key mediator of various physiological pathways, such as development, immune response, brain activity, and cancer^[6–7]^. RACK1 interacts with different proteins, especially protein kinase C (PKC). Basically, all PKCs interact with RACK proteins. The typical translocation of PKCs from cytoplasm to membrane upon activation is correlated with RACKs, especially RACK1. In addition, RACK1 preferentially interacts with PKCε^[8–9]^ and PKCβⅡ^[10–11]^, modulates their activity by stabilizing their active conformations, and facilitates their translocation near their specific substrates to activate the defined pathways^[12–13]^. Furthermore, studies have shown that RACK1 also interacts with PKCα^[14]^, PKCδ^[15]^ and PKCγ^[16]^, and recruit various proteins to form complexes. Essential cellular functions, including cell growth, proliferation, spreading, cell-cell interaction, and other similar processes, appear to be under the control of RACK1.

On the other hand, PKC isozymes found in various subcellular locations are connected to a variety of organ activities. Disparate functions of specific PKC isozyme, including regulation of ion channels, neurotransmission, synaptic plasticity, learning and memory, can be determined by the recruitment of related RACKs^[8–9]^. Similarly, numerous proteins interact with RACK1, such as the small subunit of hetero-trimeric G protein Gβ^[17]^, inositol 1,4,5-trisphosphate (IP3) receptors^[18]^, the neuronal transport protein dynamin 1^[19]^, gamma-aminobutyric acid A receptors (GABA_A_Rs)^[20]^, and N-methyl-D-aspartate receptors (NMDARs)^[21]^. By interacting with these proteins, RACK1 participates in multiple necessary neuronal functions, including intracellular Ca^2+^ regulation, protein trafficking, synaptic transmission, and plasticity^[22]^, all of which are involved in the pathogenesis of AD.

In most studies, RACK1 expression levels were significantly reduced in both membrane and cytosolic fractions of AD patient brains^[20,23]^ as well as other AD model animals, such as rats^[24]^ and rabbits^[25]^. In the aging brain, RACK1 was also reduced by approximately 50% in the membrane fraction of rats^[2,24–25]^. However, a few reports also showed no significant difference in RACK1 expression among AD patients^[26]^. Furthermore, RACK1 expression was significantly reduced in the cortex of Down syndrome patients, all of whom would develop an early-onset AD^[27]^. The down-regulated RACK1 level with aging suggests that RACK1 plays roles in the age-related diseases^[28]^. However, one study showed that overexpression of RACK1 significantly ameliorated neuronal apoptosis, blood-brain barrier disruption, brain edema, and neurological deficits after 48 h of traumatic brain injury (TBI) in rats^[27]^, demonstrating that RACK1 has the neuroprotective ability. Studies have shown that the deficit of RACK1 contributes to spatial memory impairment, which is the main characteristic of AD^[29]^, and that loss of RACK1 results in the reduction of PKC activity, which is involved in APP processing, tau hyper-phosphorylation, and inflammation in AD pathogenesis program^[28]^. Regulation of synaptic transmission is compromised, because of the dysregulated interactions between RACK1 and neurotransmitter receptors, such as the NMDARs, and GABA_A_Rs. Synaptogenesis is associated with the RACK1 or RACK1/PKC pathway. All evidence suggests that RACK1 can be involved in the pathogenesis of AD.

There are multiple ways in which RACK1 controls the progression of AD. This review examines the evidence that RACK1 interacts with and regulates a variety of important proteins based on various concepts of AD pathogenesis.

## RACK1 alleviates APP amyloidogenic processing

The incorrect cleavage of APP is an important cause of AD^[29]^. APP is a large precursor molecule that is produced broadly by neurons, blood vessels and blood cells (including the vesicles), as well as to a lesser extent by astrocytes^[30]^. In the physiological process, or the non-amyloidogenic pathway, APP is cleaved by α-secretase, such as ADAM10, to generate extracellularly released soluble APP alpha (sAPPα) and membrane-tethered C-terminal fragment (CTF) of 83 amino acids (C83). C83 is then cleaved by γ-secretase, forming the P3 peptide and intracellular domain of amyloid precursor protein^[30]^. Some studies suggest that sAPPα has a neuroprotective effect^[31–32]^ and abilities to mitigate synaptic and cognitive deficits^[33]^. In the amyloidogenic process, β-secretase first cleaves APP to produce the soluble APP beta (sAPPβ) protein and CTF, which have 99 and 89 amino acids, respectively (*i.e.*, C99 and C89). By further cleaving them, γ-secretase creates Aβ and a truncated form of Aβ, respectively^[30]^. A decrease in cognitive function is caused by neurotoxic effects of the released Aβ peptides, which cause neuronal death, apoptosis, and the loss of synapses and dendrites^[34]^. According to the amyloid hypothesis, amyloid buildup in the brain is what causes AD pathogenesis. The rest of the diseased process, including the development of tau protein-containing NFTs, is thought to be caused by an imbalance between Aβ production and clearance^[35]^.

### RACK1/PKC increases expressions of α-secretase and sAPPα

APP cleavaged by α-secretase, which has been demonstrated to be neuroprotective^[31]^, is regulated by PKC with the help of RACKs. The effect of α-secretase is specifically controlled either directly by PKCα and PKCε isozymes, indirectly by PKC activating ERK1/2, or simultaneously by both mechanisms^[36]^. The α-secretase induces the translocation of PKCα and PKCε from the cytosol to the membrane and Golgi-like structures^[36]^. The activation and translocation of PKCε isozymes is probably correlated with RACK1^[27,37]^. However, the interaction of PKCα is not clear. It was also reported that nicotine increased sAPPα expression through the RACK1/PKC pathway and that knocking down of RACK1 expression or inhibiting activation of PKC further prevented the effect of nicotine on sAPPα^[38]^. However, statins (cholesterol-lowering drugs) have been found to increase sAPP release by α-secretase activation without the participation of PKC or ERK1/2^[39]^. This means that RACK1/PKC is one of the pathways modulating α-secretase and sAPPα.

Conversely, sAPPα also regulates the expression of RACK1 and the signaling activity of PKCβⅡ by activating PI3K/Akt and nuclear factor-κB (NF-κB) pathways^[40]^. sAPPα induces PKCβⅡ translocation and increases the RACK-1/PKCβⅡ complex in the membrane^[40]^. PI3K/Akt, regulating NF-κB pathway, is one of cell survival-associated signaling pathways stimulated by sAPPα^[41]^. The expression level of NF-κB increases in the cerebral cortex of AD patients^[42–43]^. *In vitro* studies have demonstrated that sAPPα encourages C-REL nuclear translocation. Additionally, the *RACK1* promoter region has three consensus C-REL sensitive sites, demonstrating how the sAPPα-induced activation of C-REL translocation affects *RACK1* promoter activity^[7]^.

### RACK1 recruits different regulatory proteins to mediate Aβ

The formation of Aβ plaque in the cerebral cortex is a significant hallmark of AD pathology. Aβ is neurotoxic and aggravates the pathological process of AD. The 4-kDa fragment of Aβ results from the subsequent proteolytic cleavage of APP by β-secretase at the extracellular domain and γ-secretase at the intramembrane site. Initially, Aβ accumulates in cerebral regions with high metabolic bio-energetic activity rates, such as association cortices, and spreads from the neocortex to the allocortex to the brainstem, eventually reaching the cerebellum^[44]^. Studies have shown that Aβ treatment reduces RACK1 levels in the membrane fraction of prefrontal cortex neurons, and lowers total PKC levels as well as activated PKC levels in the membrane; however, RACK1 rescues the impaired GABAergic transmission caused by Aβ injection^[45]^. In mice, tanshinone ⅡA has been shown to improve Aβ_25–35_-induced spatial memory impairment by upregulating the levels of RACK1 and pERK/ERK^[46]^.

**Figure 1:**
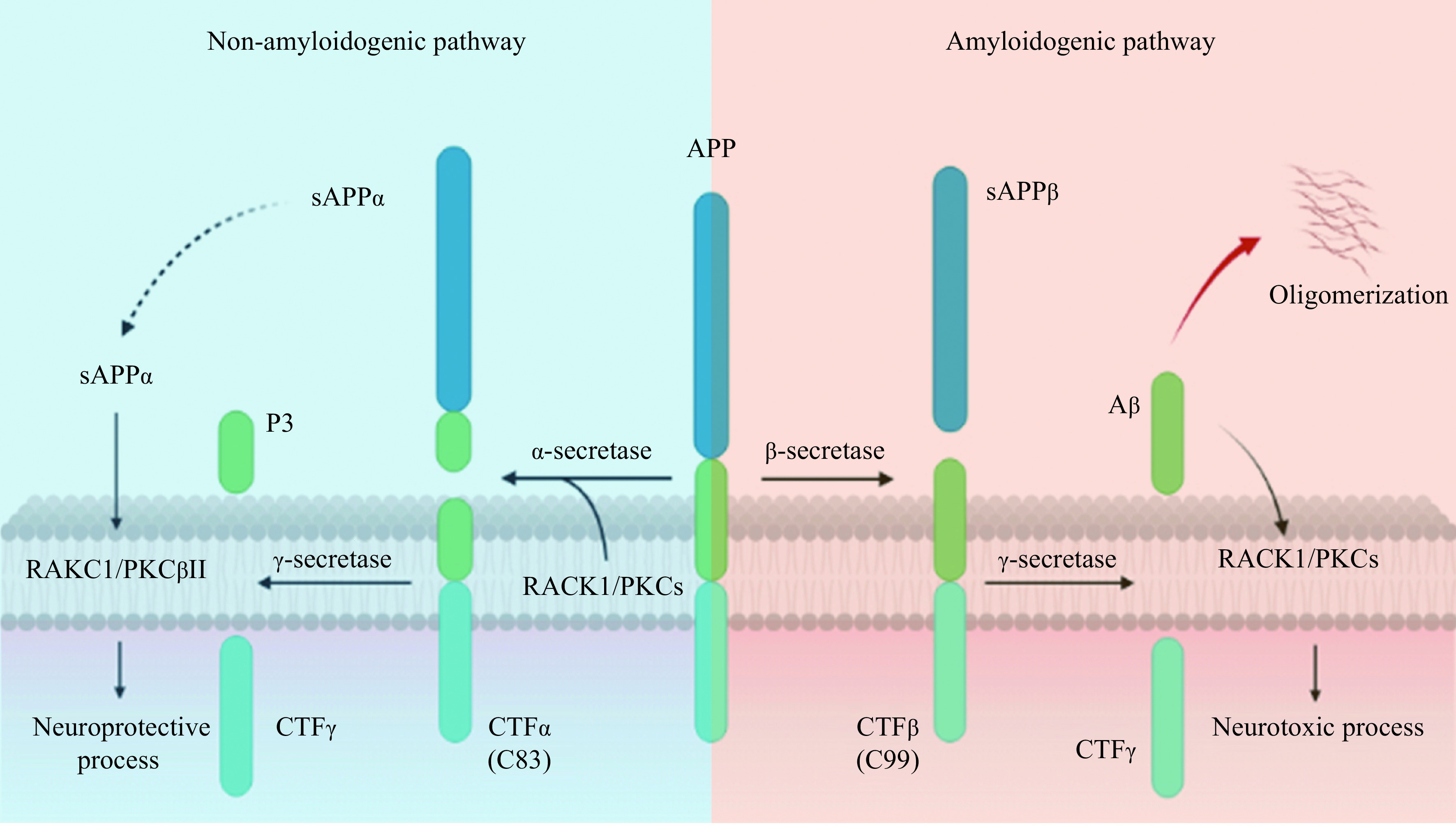
RACK1/PKCs participate in the neurofunctional pathway of APP metabolism.

Different PKC isoforms are closely correlated with the production and degradation of Aβ. Studies have shown that Aβ_1–40_ affects the activity of PKC, and Aβ_1–40_ degrades 70% of PKCγ isoforms in AD fibroblasts and 75% of PKCα in normal aged controls^[47]^, while Aβ_28–30_ residues inhibit the phorbol-12,13-dibutyrate-induced membrane translocation of PKCα and PKCε without altering their expression levels^[41]^. In addition, PKCε has been shown to reduce Aβ levels both *in vitro* and *in vivo*, and this reduction is not correlated with α-secretase but with an increase in brain endothelin-converting enzyme activity^[48–49]^. RACK1 preferentially interacts with PKCε^[8–9]^ and modulates activity by stabilizing the active conformations and facilitating their translocation near their specific substrates to activate the defined pathways^[12–13]^. Furthermore, PKCδ^[50]^ and PKCλ/ι^[51]^ have been reported to regulate β-secretase expression and Aβ production (***Fig. 1***). Inhibition of PKCδ significantly reduced the expression of β-secretase and Aβ and rescued cognitive deficits in mice^[50]^. PKC isozymes directly or indirectly regulate all pathways of APP, including post-translational processing and cleavage by α, β, or γ secretases^[52]^. Reports show that RACK1 may interact with PKCα^[14]^, PKCδ^[15]^ and PKCγ^[16]^, and recruit various proteins to form complexes. It can be speculated that RACK1 appears to be a fulcrum of cellular signals, recruiting different regulatory proteins and controlling various important cleavage processes of APP. However, the specific mechanism is still unclear.

## RACK1 and hyper-phosphorylation of tau protein

Tau phosphorylation at specific serine/threonine sites by their kinases (especially GSK3β, CDK5, and ERK2) diminishes tau's binding affinity for microtubules. This, coupled with β-amyloid peptides, culminates in the development of intracellular NFTs, representing another pivotal feature in the pathology of AD^[52–53]^. Activation of GSK3β by PKC is associated with an increase in Aβ and the development of neurofibrillary tangles. Aβ stimulates the binding of calmodulin kinase Ⅱα (CaMKⅡα) to metabotropic glutamate receptor (mGluR) 5a and acitvates ERK1/2 in an mGluR5a-dependent manner, thereby reducing tau phosphorylation and NFTs^[28,54]^. There are dual effects of PKNα and PKC on the phosphorylation of tau protein by GSK3β. PKNα and PKC directly inhibit GSK3β activity at least in part by phosphorylating Ser9 of GSK3β, and that they indirectly suppress GSK3β-stimulated phosphorylation of tau at amino acids Ser202/Thr205 and Thr181^[55]^. RACK1 is involved in the regulation of GSK3β activity in leukemic progenitors, and loss function of RACK1 may result in reduced GSK3β activity^[56]^. However, there is currently no direct evidence about RACK1 binding to the hyper-phosphorylation of tau protein.

## Role of RACK1 in synaptic transmission

### RACK1 inhibits NMDAR phosphorylation and reduces excitatory postsynaptic currents

Glutamate excitotoxicity is considered one of the core molecular mechanisms of AD neurodegeneration. NMDARs, as one of the ionotropic glutamate receptors, play a crucial role in neuronal development, excitotoxicity, synaptic plasticity, and learning and memory^[57–59]^. Functional NMDARs are composed of multiple NMDA receptor subunit 2 (NR2) and NMDA receptor subunit 1 (NR1) to form tetramers or pentamers. NR1 is the constitutive subunit of an ion channel, while NR2 is the regulatory subunit. NMDARs are composed of different NR2s showing different distributions and physiological properties in the brain^[60]^. NMDARs play their canonical roles in long-term synaptic plasticity through their Ca^2+^ permeability. In long-term potentiation (LTP), the tyrosine phosphorylation of NMDARs increases, whereas Src and Fyn are required^[61]^.

**Figure 2:**
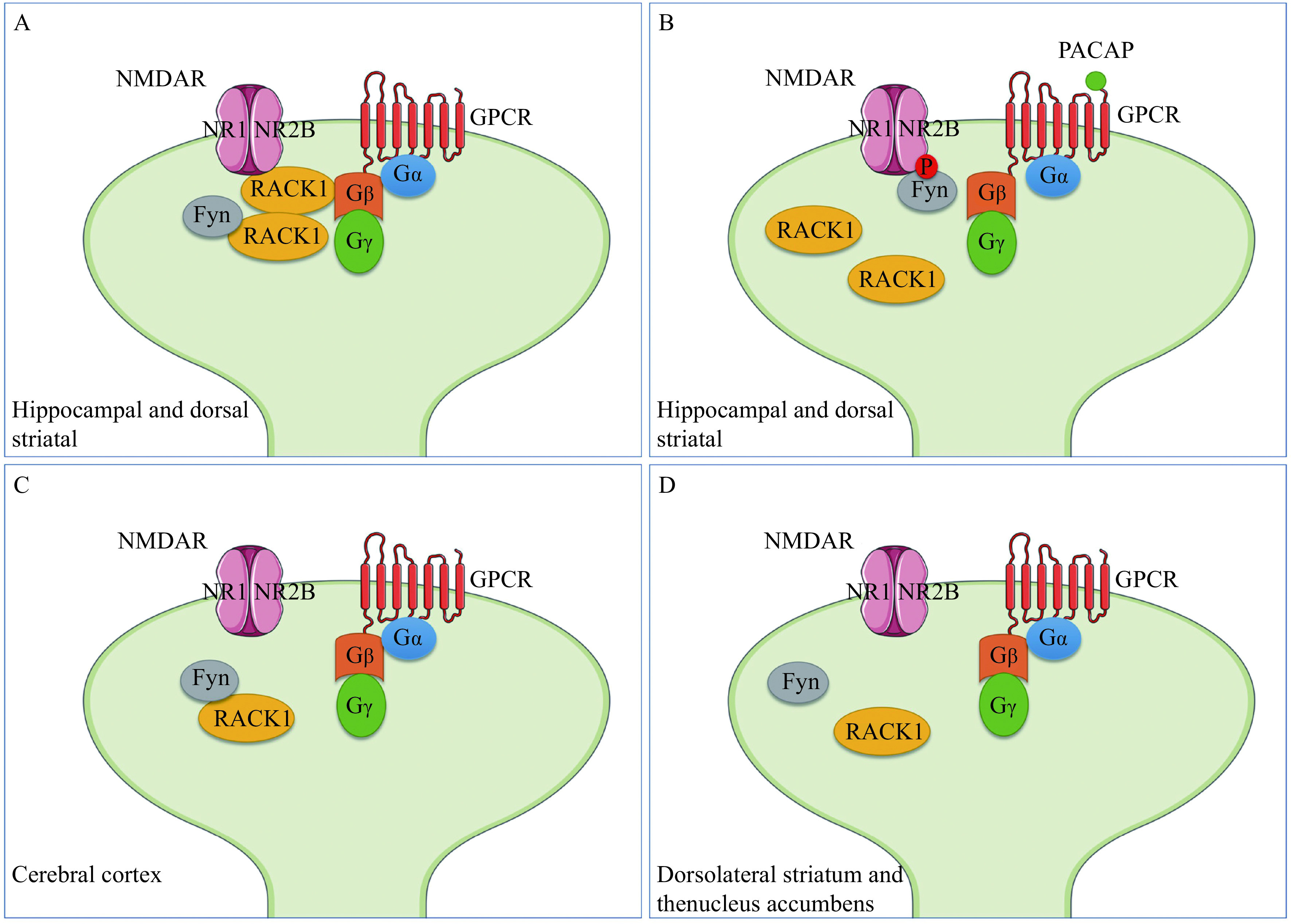
Illustration of the interactions of RACK1 and NMDARs in different brain regions.

RACK1 is essential and multifunctional in regulating the functions of NMDARs. In the hippocampus, Gβ is combined with RACK1 on the membrane to form the complex of NR2B/RACK1/Fyn at the synapse, inhibiting Fyn phosphorylation of NR2B and reducing NMDARs-mediated excitatory postsynaptic currents in the hippocampal CA1 region^[21,62]^ (***Fig. 2A***). Fyn and NR2B share homologous sequences and interact with RACK1, leading to specific RACK1 inhibition of NR2B phosphorylation by Fyn. The interaction between RACK1 and NR2B prevents the interaction of Fyn and NR2B active sites^[21]^. However, no homology sequence shared between Fyn and ctNR2B was found in the NR1, NR2C, and NR2D receptor subunits. Activation of the cyclic AMP (cAMP)/PKA pathway by pituitary adenylyl cyclase-activating protein or alcohol exposure causes dissociation of Gα from Gβγ. Free Gβγ has a reduced affinity for RACK1, destabilizing the trimolecular complex. RACK1 must be removed from the NMDARs for Fyn to be free to bind to phosphorylate tyrosine residues on cytoplasmic tail of NR2B, which in turn increases the channel's activity^[21,62–63]^ (***Fig. 2B***). In the cerebral cortex, RACK1 binds to Fyn but not NMDARs, although these three proteins are expressed throughout the brain^[21–22,63–64]^ (***Fig. 2C***). In the ventral striatum, GluN2B is phosphorylated by the activated Fyn only in the dorsomedial striatum, but not in the dorsolateral striatum and the nucleus accumbens^[65]^ (***Fig. 2D***).

RACK1, acting as a scaffold for PKC, mediates Ca^2+^ release by enhancing IP3 receptor binding affinity for IP3^[18]^. The regulation of Ca^2+^, which is crucial for LTP in nerve cells, is primarily controlled by IP3 receptors^[66]^. A single train of action potentials initiates early LTP by activating NMDARs, facilitating the Ca^2+^ influx into postsynaptic cells and promoting receptor activation and interaction with the Src family kinase Fyn. Both NMDARs and Fyn interact directly with RACK1, implying that RACK1 is causally involved with specific LTP processes. The PKCs, interacting with RACK1, regulate the gating and trafficking of NMDAR channel in the neurons, which is the mechanism intimately associated with synaptic plasticity, while the dysregulation of PKC by RACK1 in hippocampal neurons plays an important role in memory deficits of aging cells^[23]^.

### RACK1/PKC-βⅡ activates GABA_A_Rs

The imbalance between excitation and inhibition significantly contributes to the pathology of AD^[67–68]^. GABA is the primary inhibitory transmitter in the adult mammalian brain, and the elevated GABA levels have been observed in the cerebrospinal fluid of AD patients^[69]^. GABA_A_Rs are the primary inhibitory receptors in the central nervous system and are assembled from five subunit classes: α, β, γ, δ, and ε. The receptor function is modulated by direct phosphorylation of the β and γ2 subunits. In AD model mice, the administration of GABA_A_R antagonists significantly improves long-term potentiation in the hippocampus^[70]^.

In the central nervous system, RACK1 binds not only to NMDARs but also to cytoplasmic domains of the β1 and β3 subunits of GABA receptors^[20,71]^. RACK1 promotes the phosphorylation of GABA_A_R by PKCβⅡ, thereby regulating GABA_A_R function. RACK1/PKC-βⅡ is also able to bind to the intracellular domains of the β1 and β3 subunits of the receptors but not to those of the α1 or γ2 subunits^[20]^. PKC-βⅡ specifically phosphorylates serine 409 in the β1 subunit and serine 408/409 in the β3 subunit, which are key residues in function regulation of GABA_A_Rs. PKC activation inhibits the function of GABA_A_Rs in the cortical neurons, whereas inhibition of PKC activity inversely increases GABA-activated currents and decreases phosphorylation of the β3 subunit^[20,72]^. Therefore, there is a negative feedback regulation between RACK1/PKC-βⅡ and GABA_A_R activation. In the prefrontal cortex pyramidal neurons, serotonin receptors regulate GABA_A_R channels by activating RACK1^[73]^. Targeting the activated PKC to GABA_A_Rs by RACK1 is critical for regulating GABA_A_ currents through the 5HT2-PKC signaling pathway. A large number of compounds that interact with GABA receptors, such as neurosteroids^[74]^, alcohol^[75]^ and sodium valproate^[76]^, work through the RACK1/PKC-βⅡ-dependent pathway. In the prefrontal cortex, Aβ impairs GABAergic transmission mediated by muscarinic receptors; however, overexpression of RACK1 rescues this damage^[45]^.

### RACK1 promotes synaptogenesis and synaptic plasticity

Synaptic plasticity is a crucial mechanism for maintaining memory, which involves the growth of new synaptic connections or pruning of existing ones, modification of the strength or efficacy of synaptic transmission, and modulation of the excitability of existing synapses. Structural and functional plasticity work together, leading to synaptic plasticity that induces structural modifications in dendritic spines, promoting spine head growth, formation, and maintenance, and inducing LTP^[77]^. Neurodegenerative diseases, such as AD, show marked changes in synaptic structure and function^[77–78]^. A number of studies have shown that RACK1 is involved in brain synapse formation and synaptic structural plasticity^[79–81]^. In RACK1 cKO mice, synaptogenesis, synaptic transmission, and LTD were impaired, while the synaptic protein levels PSD95, GAD65, Dynamin1, and Synapsin remained unchanged^[82]^. RACK1 also directly regulates axonal growth, while the expression of RACK1, either too high or too low, affects the growth of axons, but knockdown or overexpression of RACK1 has resulted in axonal shortening and abolished brain-derived neurotrophic factor (BDNF)-induced increase in growth cone area^[80]^. This demonstrates how important RACK1 expression levels are for the axon development and neurogenesis.

RACK1 is required for synaptic contact formation and is a key member of synaptic contact complex^[79]^. For example, knockdown of *RACK1* with shRNA decreased the transcription and expression of BDNF^[83]^. The non-phosphorylated mutant form of RACK1 also abolished the effect of BDNF stimulation in point contact density^[83]^. In neurons, the growth of synaptic axes is inseparable from the local translation of β-actin mRNA within the growth cone to guide properly, which requires RACK1 expression and phosphorylation^[79]^. The release and translation of β-actin mRNA depend on Src kinase phosphorylation-regulated Zipcode-binding protein 1 (ZBP1) expression. RACK1 also binds to the β-actin mRNA/ZBP1 complex on the ribosome and causes Src phosphorylation, promoting the release and translation of β-actin mRNA^[84]^. Furthermore, RACK1 anchors Arf-GAP with GTPase, ANK repeat, and PH domain 2 to focal adhesion kinase, thereby regulating neurite outgrowth, which is reduced when these interactions are disrupted^[85]^.

## Inflammatory reactions and RACK1

### RACK1 regulates NF-κB by activating PKCβⅡ

Besides Aβ plaques, inflammation stigmata and oxidative stress are both featured lesions found in the brains of AD patients^[86]^. These immune system-mediated actions promote AD pathogenesis^[87]^. Oxidative stress and inflammation are characterized by the release of cytokines and reactive oxygen species, known to activate NF-κB^[88]^. NF-κB is a stress-activated transcription factor to be activated around senile plaques and has binding sites in the promoter region of the genes involved in amyloidogenesis and inflammation^[86]^. In addition to activating the transcription of *APP*, β-secretase and some of the γ-secretase members, NF-κB also enhances the protein expression and enzyme activities, resulting in the enhanced Aβ production^[89]^.

The interaction of intracellular acetylcholinesterase-S on NFκB prevents NFκB from being activated by residual RACK1 and its interacting protein kinase PKCβⅡ^[90]^. RACK1 recruits its partner proteins and interacts with various cytoplasmic proteins and transmembrane receptors, thus providing a platform for subcellular mobilization and subsequent physiological responses. The RACK1 region supporting acetylcholinesterase receptor (AChE-R) interaction consists of 30% of the RACK1 perimeter. RACK1-AChE-R interactions and PKC activators, which modulate RACK1-PKCβⅡ interactions, may therefore compete with other RACK1 associations, changing the subcellular balance between different RACK1-containing complexes under stress^[91]^. Overexpression of AChE-R promotes synaptic plasticity and enhances fear memory through RACK1 and PKCβⅡ^[92]^. In the lipopolysaccharide (LPS)-induced inflammatory injury model of mouse microglia BV2 cells, miR-155 knockdown deactivated MAPK/NF-κB and mTOR signaling pathways by targeting RACK1 for cell protection^[93]^.

### Pro-inflammatory cytokines and RACK1

During AD progression, both Aβ and microglial activation may lead to the production and release of pro-inflammatory cytokines, such as IL-1β, IL-6, IL-18, TNF-α, IL-10, and TGF-β^[4]^. Most of these pro-inflammatory factors, given IL-1β^[94]^, IL-6^[94]^, IL-18^[95]^ and TNF-α^[96]^ as examples, are reported to interact with RACK1. The reduction of RACK1 abrogates caspase-1 activation and IL-1β release in response to the NLRP3 stimuli^[97]^. The expression levels of LPS-induced IL-8, TNF-α, and CD86 are regulated by RACK1/PKCβ in THP-1 cells and primary human dendritic cells. For example, a selective inhibitor of PKCβ completely blocked the LPS-induced CD86 expression and resulted in a 50% reduction of IL-8 and TNF-α release; in contrast, the RACK1 pseudo substrate directly activated PKCβ and concentration-dependently increased CD86 expression and IL-8 release^[98]^. PKC isozymes also regulate the levels of TNF-α and IL-6 and the release of other cytokines in the brains of AD patients by phosphorylating MAPK, Erk1, and Erk2^[99]^. In addition, RACK1 suppresses the TNF-α-induced cell death in L929 cells by enhancing p38 activation^[96]^ and thus plays an essential role in TNF-α-induced inflammatory and cell death.

### RACK1 mediates NLRP3 inflammasome activation

The NLRP3 inflammasome is a key component of the innate immune system, which is important in the pathogenesis of AD. NLRP3 inflammasome induces caspase-1 activation and IL-1β maturation in response to infection or cellular damage. The Aβ-mediated activation of the NLRP3 inflammasome in microglia leads to IL-1β maturation and subsequent inflammatory events^[100]^. RACK1 also plays a crucial role in NLRP3 inflammasome activation. Both NLRP3 and its interacting partner NEK7 interact with RACK1, and RACK1 promotes NLRP3 activation, while reduction of RACK1 expression inhibits caspase-1 activation and IL-1β release in response to NLRP3^[97]^. In macrophage, RACK1 forms a complex with EST12, which recruits the deubiquitinase UCHL5 to promote the K48-linked deubiquitination of NLRP3, resulting in NLRP3 inflammasome activation, caspase-1/11-dependent pyroptosis and gasdermin D-IL-1β immune response^[101]^.

### RACK1 is correlated with microglia activation

Microglia, the resident immune cells in the central nervous system (CNS), play a crucial role in removing pathogenic elements and promoting tissue repair^[102]^. However, in the context of AD, microglial activation appears to exacerbate disease pathology^[103]^. Studies have shown a marked increase in microglial activation in the brains of AD patients, which may contribute to neuroinflammation and neuronal cell death^[103]^.

RACK1 is also involved in the microglial activation, affecting their inflammatory responses^[104]^. RACK1 knockdown in the microglia has been found to decrease the production of pro-inflammatory cytokines in response to LPS, a bacterial endotoxin that is commonly used to activate the microglia *in vitro*^[104]^. While RACK1 is also involved in cell polarity^[105]^ and chemotaxis^[106]^ in tumor cells, there is no direct evidence that RACK1 regulates polarity and chemotaxis of the activated microglia. In the APP/PS1 model of AD, the deficiency of the NLRP3 inflammasome results in a decreased Aβ deposition and skewed microglial cells to an M2 phase^[100]^.

## RAKC1 and androgens

Aging is a major risk factor for AD, and one of the most noticeable changes that occur with age is the decreased hormone levels, especially sex hormones, such as dehydroepiandrosterone (DHEA) and dihydrotestosterone, which are important androgens. They may all be associated with cognitive impairment by activating enzymes involved in memory processes. Androgen deficiency because of aging is a major cause of cognitive impairment, and both a decreased serum testosterone level and an increased luteinizing hormone level are risk factors for AD in men with dementia, which may be negatively regulated by Aβ^[97,101]^.

Age-related declines in RACK1 expression and PKC-dependent activities have been associated with a decreased DHEA secretion. *In vitro* and *in vivo* DHEA injections have been found to return RACK1 expression to the baseline levels in cells from elderly animals and humans^[107–108]^. DHEA works by binding to the androgen receptor (AR), and RACK1 may function as a scaffold for the association and modification of AR by PKC, enabling translocation of AR to the nucleus but rendering AR incapable of activating transcription of its target genes^[107]^. Complete blockade of *AR* expression by siRNA prevented DHEA-induced *RACK1* mRNA expression^[109]^. Administration of DHEA has been shown to rescue age-reduced RACK1 expression, LPS-induced IL-8 release, and TNF-α production^[108]^. DHEA also controls RACK1 expression by regulating GRβ expression, and total knockdown of GRβ blocks the DHEA-induced RACK1 expression and cytokine release modulation, while DHEA produces a dose-dependent upregulation of GRβ^[110]^. These contribute to the effect of DHEA as an anti-glucocorticoid on the immune system^[111]^. The complex hormonal balance between glucocorticoids and androgens regulates RACK1 expression, because of the specificity and intricate interactions on the RACK1 promoter.

## RACK1 prevents neuronal apoptosis

RACK1 exerts neuroprotective effects by preventing neuron death in brain injuries, such as TBI and cerebral ischemia-reperfusion. Overexpression of RACK1 significantly ameliorates neuronal apoptosis, blood-brain barrier disruption, brain edema, and neurological deficits after TBI, possibly mediated by activation of the IRE1-XBP1 pathway^[26]^. At the same time, the up-regulation of RACK1 reduces infarct size, neuronal death, neuronal tissue loss, and neurobehavioral dysfunction in cerebral ischemia-reperfusion injury^[112]^. In addition, RACK1 participates in the formation of autophagosome biogenesis complex upon its phosphorylation by AMPK at Thr50. Thr50 phosphorylation of RACK1 enhances its direct binding to VPS15, ATG14L, and beclin 1, thereby promoting the assembly of the autophagy-initiation complex^[113]^. Finally, RACK1 promotes self-renewal and chemoresistance of human liver cancer stem cells, and maintains functions of murine embryonic stem cells^[114]^. These neuroprotective effects of RACK1 may be potential mechanisms that delay the pathological process of AD.

## Conclusions and perspectives

RACK1 is abundantly expressed in the CNS, particularly in neurons, and localizes to neuronal cell bodies and dendrites throughout the developing brain^[115]^. Loss of RACK1 dysregulates the localization and distribution of different PKC isoforms, subsequently influencing specific neural function^[23]^. In AD, RACK1 directly or indirectly regulates the most related pathways of APP, including amyloidogenic and non-amyloidogenic pathway processing, which are regulated by the cleavage of α- or β-secretase. Besides, RACK1 inhibits NMDARs and active GABA_A_Rs, thus preventing neuronal excitotoxicity. RACK1 also promotes synaptogenesis and synaptic plasticity. On the other hand, RACK1 regulates inflammatory and cell death. Taken together, RACK1 plays a crucial role in multiple aspects of AD pathogenesis and regulates different physiological or pathological processes by binding to different membrane proteins.

The RACK1-regulated cAMP and PKC signaling pathways mediate a wide range of cellular processes, such as cell proliferation and differentiation, metabolism, and inflammation. The cyclic AMP phosphodiesterase (PDE) degrades the second messenger cAMP to 5′-AMP. The most dominant PDE activity of inflammatory cells is PDE4, which reduces 3′,5′-cAMP levels in the CNS and thereby regulates the PKA activity and the phosphorylation of CREB. These processes are fundamental to depression, cognition, and learning and memory. PDE4D5 isoforms, expressed about three times higher in the cell membrane fraction, have been found to interact with the ubiquitously expressed WD40 signaling scaffold protein RACK1^[116]^. RACK1 may recruit PDE4D5 and PKC to intracellular proteins to control cellular cyclic AMP levels^[117]^. The location and action of PDEs are regulated by the interactions with RACK1, which may form multi-component complexes, including RACK1, AChE-R, and their PKC or PDE4 partners^[81]^.

In conclusion, RACK1 has an extensive and significant association with AD as a potential therapeutic target. Although the role of RACK1 in AD remains to be fully understood, continued research will ultimately lead to new insights and potential therapies.
